# Efficient generation of P53 biallelic knockout *Diannan* miniature pigs via TALENs and somatic cell nuclear transfer

**DOI:** 10.1186/s12967-017-1327-0

**Published:** 2017-11-03

**Authors:** Youfeng Shen, Kaixiang Xu, Zaimei Yuan, Jianxiong Guo, Heng Zhao, Xuezeng Zhang, Lu Zhao, Yubo Qing, Honghui Li, Weirong Pan, Baoyu Jia, Hong-Ye Zhao, Hong-Jiang Wei

**Affiliations:** 1 State Key Laboratory for Conservation and Utilization of Bio-Resources in Yunnan, Yunnan Agricultural University, Kunming, 650201 China; 2 College of Animal Science and Technology, Yunnan Agricultural University, Kunming, China; 3 Key Laboratory Animal Nutrition and Feed of Yunnan Province, Yunnan Agricultural University, Kunming, 650201 China

**Keywords:** P53, TALENs, SCNT, *Diannan* miniature pig

## Abstract

**Background:**

Pigs have many features that make them attractive as biomedical models for various diseases, including cancer. P53 is an important tumor suppressor gene that exerts a central role in protecting cells from oncogenic transformation and is mutated in a large number of human cancers. P53 mutations occur in almost every type of tumor and in over 50% of all tumors. In a recent publication, pigs with a mutated P53 gene were generated that resulted in lymphoma and renal and osteogenic tumors. However, approximately 80% of human tumors have dysfunctional P53. A P53-deficient pig model is still required to elucidate.

**Methods:**

Transcription activator-like effector nucleases (TALENs) were designed to target porcine P53 exon 4. The targeting activity was evaluated using a luciferase SSA recombination assay. P53 biallelic knockout (KO) cell lines were established from single-cell colonies of fetal fibroblasts derived from *Diannan* miniature pigs followed by electroporation with TALENs plasmids. One cell line was selected as the donor cell line for somatic cell nuclear transfer (SCNT) for the generation of P53 KO pigs. P53 KO stillborn fetuses and living piglets were obtained. Gene typing of the collected cloned individuals was performed by T7EI assay and sequencing. Fibroblast cells from *Diannan* miniature piglets with a P53 biallelic knockout or wild type were analyzed for the P53 response to doxorubicin treatment by confocal microscopy and western blotting.

**Results:**

The luciferase SSA recombination assay revealed that the targeting activities of the designed TALENs were 55.35-fold higher than those of the control. Eight cell lines (8/19) were mutated for P53, and five of them were biallelic knockouts. One of the biallelic knockout cell lines was selected as nuclear donor cells for SCNT. The cloned embryos were transferred into five recipient gilts, three of them becoming pregnant. Five live fetuses were obtained from one surrogate by caesarean section after 38 days of gestation for genotyping. Finally, six live piglets and one stillborn piglet were collected from two recipients by caesarean section. Sequencing analyses of the target site confirmed the P53 biallelic knockout in all fetuses and piglets, consistent with the genotype of the donor cells. The qPCR analysis showed that the expression of the P53 mRNA had significant reduction in various tissues of the knockout piglets. Furthermore, confocal microscopy and western blotting analyses demonstrated that the fibroblast cells of *Diannan* miniature piglets with a P53 biallelic knockout were defective in mediating DNA damage when incubated with doxorubicin.

**Conclusion:**

TALENs combined with SCNT was successfully used to generate P53 KO *Diannan* miniature pigs. Although these genetically engineered *Diannan* miniature pigs had no tumorigenic signs, the P53 gene was dysfunctional. We believe that these pigs will provide powerful new resources for preclinical oncology and basic cancer research.

**Electronic supplementary material:**

The online version of this article (10.1186/s12967-017-1327-0) contains supplementary material, which is available to authorized users.

## Background

The key tumor suppressor gene P53 plays an important role in a wide range of cellular processes, including apoptosis, cell cycle arrest, senescence, energy metabolism, and anti-oxidant defense [1]. These stress signals stimulate the activation of P53 protein, which is mediated largely through the activity of P53 in transcriptional regulation of its target genes [2–4]. Transactivation-independent activities of P53 have also been described, ranging from transcriptional repression to cytoplasmic and mitochondrial functions [5, 6]. P53 is the most commonly inactivated gene in sporadic human cancers [7]. It was estimated that approximately 80% of human tumors have dysfunctional P53. P53 mutations occur in almost every type of tumor and in over 50% of all tumors [8]. Germline P53 mutations in humans cause Li-Fraumeni syndrome, a familial condition characterized by early onset of different tumors [9, 10]. Moreover, the P53 gene is somatically mutated or deleted in a large number of human cancers, indicating that this tumor suppressor exerts a protective role against oncogenic transformation in multiple tissues [11].

Many P53 modifications have been generated in mice, including knockout and inducible oncogenic activation mutations [12]. Although genetically engineered mouse models have significantly contributed to cancer biology [13], they still have significant limitations in their usefulness for modeling human cancer due to the differences in human and mouse biology [14, 15]. Since the physiology, anatomy, pathology, genome organization, body weight, and life spans of pigs and miniature pigs are more similar to those of humans, the pig represents an excellent biomedical model compared to rodents for specific human diseases, including cancer [16, 17]. Recently, the porcine P53 gene was mutated by the introduction of missense mutations via rAAV, and pigs with lymphoma and renal and osteogenic tumors were generated [18, 19]. However, a porcine P53 deficiency pigs model is still required to elucidate.

The gene targeting efficiency of traditional DNA homologous recombination (HR) technology is extremely low [20]. In many cases, additional cloning or breeding steps are required to produce biallelic mutant animals due to Cre or virus-related vectors commonly inducing DNA single-strand mutations [19, 21]. The transcription activator-like effector nucleases (TALENs) provide a highly efficient and precise means for gene targeting by introducing double-strand breaks (DSB) at preselected sites [22–24]. TALENs has great promise for creating genetically engineered pigs [22, 25, 26]. Recently, we generated GGTA1 knockout *Diannan* miniature pigs and the MSTN knockout small tail Han sheep by combining TALENs with SCNT [27, 28].

In this study, we generated genetically modified *Diannan* miniature pigs via gene editing in the somatic cells of *Diannan* miniature pigs using TALEN technology followed by SCNT to produce P53 KO *Diannan* miniature pigs. Phenotypic characterization of the mutated pigs was also performed. These genetically engineered *Diannan* miniature pigs will provide a powerful new resource for preclinical oncology and basic cancer research.

## Methods

### Chemicals

Unless otherwise stated, all chemicals were purchased from Sigma Chemical Co. (St. Louis, MO, USA).

### TALEN design and generation

TALENs targeting exon 4 of the porcine P53 gene (ensemble ID: ENSSSCG00000017950) were designed and assembled by ViewSolid Biotech Company (Beijing, China). The P53 TALENs recognition sequences were as follows: left TALEN 5′-TCTGGAACAGCCAAGT-3′ and right TALEN 5′-CCCTCAAGGCCACTGAC-3′. The coding region of TALENs with ForkI was cloned into pCAG vectors. The targeting efficiency of TALEN vectors in vitro was evaluated by a luciferase single strand annealing (SSA) recombination assay as described previously [28].

### Cell culture, transfection and selection

Pig fetal fibroblasts (PFFs) were prepared as previously described [29]. Prior to transfection, the PFFs were thawed and cultured in medium (10% FBS and 1% PS) until sub-confluence was reached. Approximately 7 × 10^5^ PFFs in 700 μL PBS mixed with 10.5 μg of the TALEN plasmid pairs were transfected by electroporation at 250 V for a single 20-ms pulse (Gene Pulser Xcell Microbial System, Bio-Rad, USA) in a 4-mm gap cuvette. Then, the cells were seeded in 5 mL of fresh DMEM containing 10% FBS in a T25 culture flask following a 48-h incubation at 37 °C. The cells were then trypsinsed, and the extremely dilute culture method was used to cultivate the cells and obtain single cell colonies. After 12–14 days, the colonies were assessed via polymerase chain reaction (PCR) (upstream primer, 5′-ACTGCTCTCTGCCCTTGTCTT-3′; downstream primer, 5′-AGAGTGTGATGGGAAGGATGAG-3′), and the amplified fragments were used for genotyping, including restriction endonuclease analysis and sequencing. Finally, we selected positive fibroblasts cell lines with a biallelic KO as nuclear donors for SCNT.

### In vitro maturation of oocytes

Oocyte collection and culture were performed as previously described [29].

### SCNT and generation of P53 KO fetuses and piglets

Somatic cell nuclear transfer (SCNT) was performed as previously described [29]. P53 biallelic knockout fibroblasts (donor cells) were cultured to sub-confluence before nuclear transfer and then injected into the perivitelline space of an enucleated oocyte. The donor cell membrane should be in contact with the oocyte cytoplasmic membrane. Oocyte cytoplasm-cell complexes were then fused and activated by electric pulses. The normal cleavage and blastocyst stages of the oocytes were recorded at 2 and 7 days of culture, respectively. The blastocyst cell number was counted under an ultraviolet light microscope after fixing and Hoechst 33342 staining. Reconstructed embryos cultured for 14 or 16 h were surgically transferred into five crossbred (large white/landrace duroc) recipient gilts the day after observed estrus. One pregnancy was delivered by cesarean section on day 38 of gestation to establish fetal fibroblast cell lines and genotyping. Pregnancy was confirmed at approximately 23 days after surgical transfer using an ultrasound scanner (HS-101V, Honda Electronics Co., Ltd., Yamazuka, Japan). Fetuses and piglets were recovered with cesarean surgery at different developmental stages. The deliveries were performed by cesarean section on day 111 or 112 of gestation. *Diannan* miniature pigs of the same age produced by normal sexual reproduction were used as controls.

### Detection of gene mutations

The genomic DNA of each cell colony, fetus and ear tissues from each newborn cloned piglet was extracted with the TIANamp genomic DNA kit (Tiangen, Beijing, China). Mutations in P53 were assessed using PCR followed by T7 endonuclease I (T7EI) digestion. The genotyping primers for P53 were as follows: upstream primer, 5′-ACTGCTCTCTGCCCTTGTCTT-3′ and downstream primer, 5′-AGAGTGTGATGGGAAGGATGAG-3′. Briefly, to identify the sequence knockout by TALENs, the PCR products of 19 cell colonies were digested by T7EI. The positive sequence that had not been digested was purified using a gel extraction kit to prepare for cloning using a recombined plasmid with the PMD18-T plasmid vector (Takara) that was sequenced to determine the exact mutant sequences (Sangon Biotech Co., Ltd., Shanghai, China). DNA mutations were identified by sequence alignment between the sequenced allele and the wild-type (WT) allele. Mutation frequencies were calculated as previously described [23]. Cell colonies harboring mutations were cryopreserved for SCNT.

### RNA isolation and qPCR

Various tissues, including heart, liver, spleen, lung, kidney, muscle and brain were obtained from P53 KO and WT piglets, frozen immediately in liquid nitrogen and stored at − 80 °C until use. The total RNA of the tissues and cultured cell treatment with and without 100 μM of DOX for 24 h was isolated using TRIzol (Invitrogen, USA) according to the manufacturer’s instructions. cDNA was synthesized from total RNA using a Super RT Kit (TakaRa, Dalian, China). The obtained cDNA was used as a template in SYRB green-based q-PCR (CFX-96, Bio-Rad, USA). The primer sequences can be found in Additional file 1: Table S1. The mRNA expression levels of the P53 were assessed by quantitative-polymerase chain reaction (q-PCR). GAPDH was used for normalization.

### Protein extraction and immunoblotting

FFCs were cultured at a density of 1 × 10^6^/well in a 10-cm plate and treated with or without DOX (100 μmol/L) for 24 h. The cells were harvested and lysed in RIPA lysis buffer (Beyotime, China) with protease inhibitors at 4 °C. After lysis, supernatants were obtained by centrifugation at 14,000×*g* for 15 min at 4 °C. The proteins (50 μg) were separated using SDS-PAGE. After electrophoresis, the proteins were transferred to polyvinylidene difluoride (PVDF) membranes and reacted with primary antibodies against P53 (Imaxgen), P21 (Eptomics) and GAPDH (Sigma). After incubation, membranes were washed and incubated with anti-rabbit secondary antibodies (R&D, USA). The membranes were developed using the ECL detection system (Easysee Western Blot Kit, China) and visualized with an imagining system (Bio-Rad, Universal Hood II, USA).

### Confocal fluorescence microscopy

Fibroblasts cells of P53 KO and WT piglets (7.5 × 10^4^) cultured in the medium with or without 100 µM DOX for 24 h were fixed in 4% (w/v) paraformaldehyde overnight at 4 °C and permeabilized with 0.05% Triton X-100 for 30 min. Then, the cells were incubated with an antibody against P53 at 4 °C overnight. After washing with PBS three times, the cells were incubated with 400-fold diluted Alexa Fluor 488-labeled anti-mouse IgG (Thermo Fisher). The nuclei of the incubated cells were stained with 5 μg/mL Hoechst 33342, and the cells were observed using confocal fluorescence microscopy (FV1000, Olympus Corporation).

### Statistical analysis

All values were expressed as the mean ± SD, and they were statistically analyzed by Student’s t test using GraphPad Prism 5 software (La Jolla, CA). *p < 0.05 and **p < 0.01 versus the control were considered as statistically significant.

## Results

### Construction of TALENs targeting P53 gene and validation of activity

**Fig. 1:**
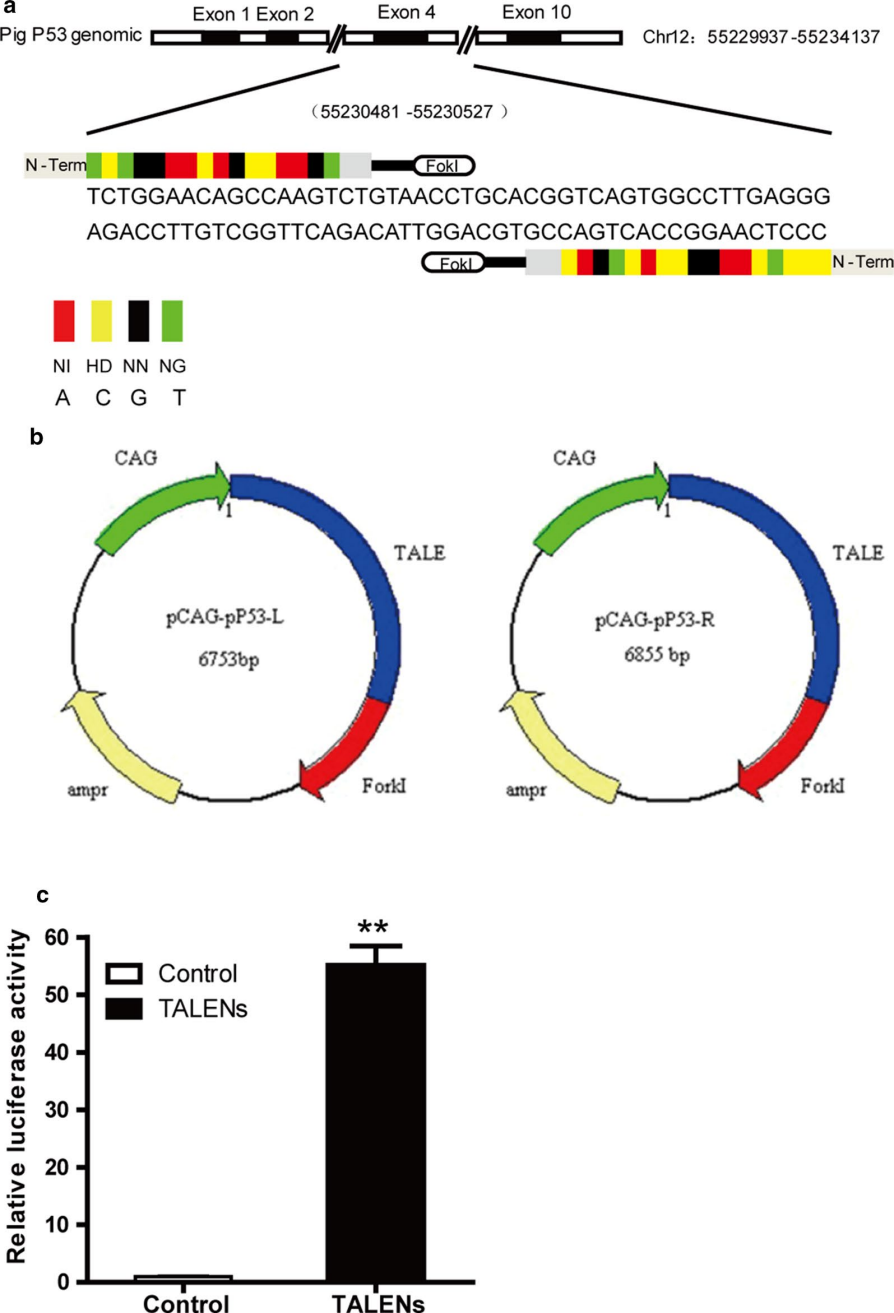
TALEN design and activity. **a** A schematic diagram of TALEN targeting exon 4 of the porcine P53 gene. **b** A schematic diagram of TALEN plasmids specific for P53 gene. **c** The detection of TALEN activity using a luciferase SSA recombination assay. Luciferase activity was increased by 55.35-fold compared to the control activity

TALEN pairs targeting exon 4 of porcine P53 were commercially synthesized; the constructs were shown in Fig. 1a, b. The activity was validated using the luciferase SSA recombination assay. The luciferase activity of the TALEN pairs targeted-P53 gene was increased 55.35-fold compared to the activity of control (Fig. 1c), indicating that the TALEN pairs were capable of recognizing their target DNA sequences. Thus, the pair of TALEN plasmids that recognized exon 4 of P53 was used to generate gene-targeted *Diannan* miniature pig fetal fibroblast cells.

### Generation of P53 mutant *Diannan* miniature pig fetal fibroblast cells

**Fig. 2:**
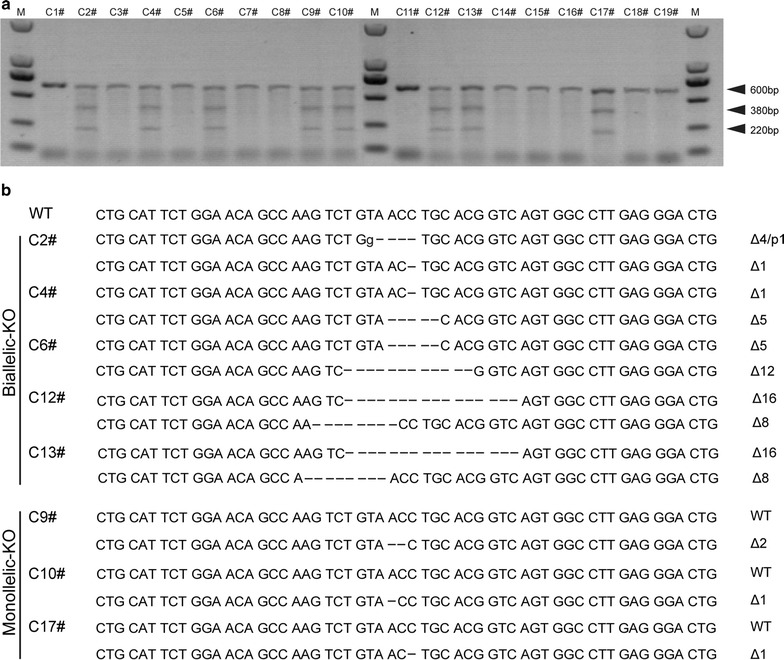
TALEN-mediated P53 mutations in PFFs. **a** Identification of P53-mutant cell lines. Cell lines showing one band indicate the WT allele, while mutated alleles produced three bands in a T7EI assay. **b** The sequences of P53-mutant cell lines. The WT sequence is shown above. Deletion and point mutations (denoted with “Δ” and “p” with the number of base pairs) are identified

The TALEN plasmids were transfected into *Diannan* miniature pig fetal fibroblast cells via electroporation. After 2 weeks of culture, eight positive colonies were obtained from a total of 19 cell colonies by T7EI assay (Fig. 2a). Then, the positive colonies were genotyped by sequencing, which showed that three were monoallelic KO and five were biallelic KO (Fig. 2b). The results showed that the TALEN-mediated targeting efficiency was up to 42%, and five mutant colonies were biallelic KO.

### Generation of P53 knockout fetuses and piglets

**Fig. 3:**
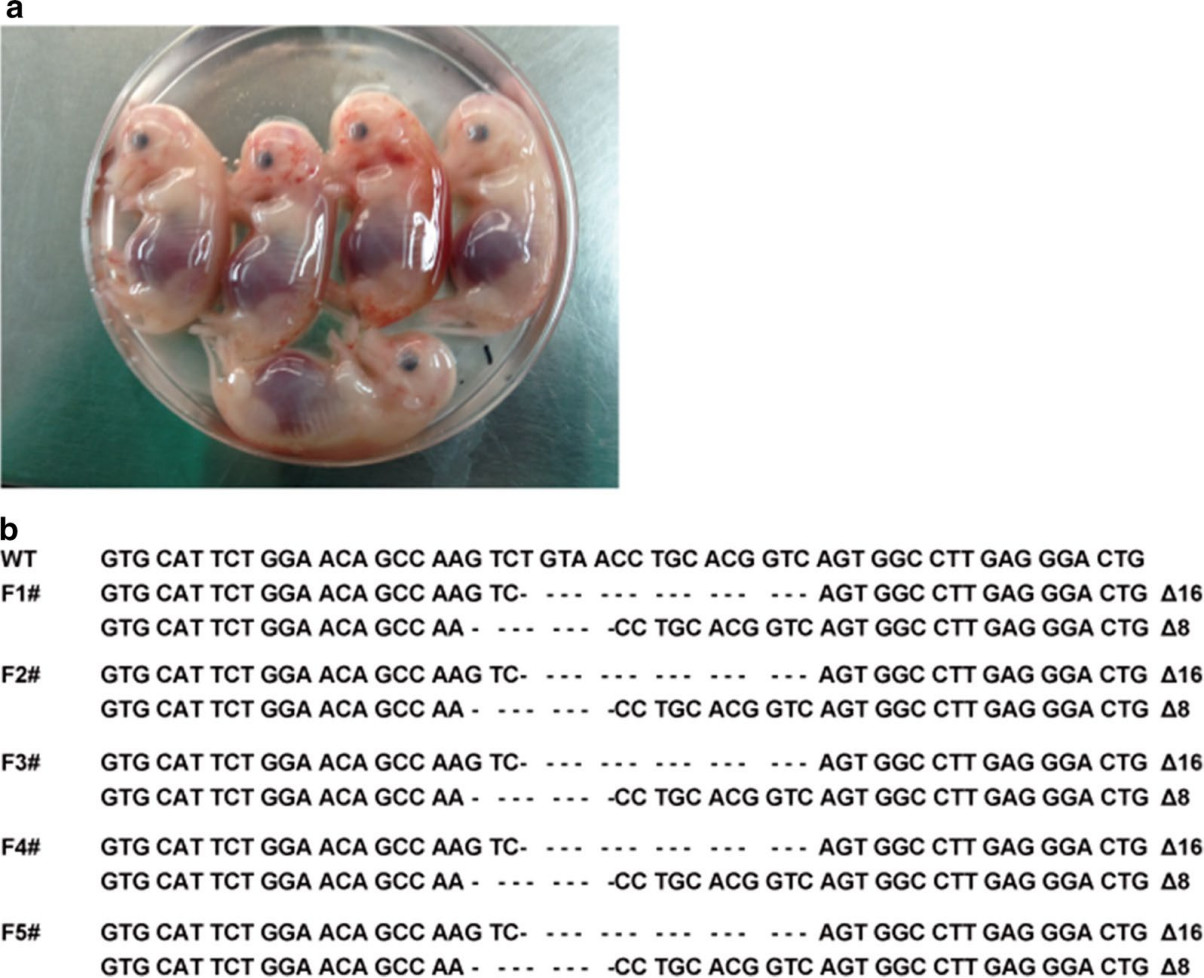
TALEN-mediated P53 mutations in cloned fetuses. **a** Five P53 KO live fetuses were obtained after 38 days of gestation. **b** The sequences of the P53 mutation in the fetuses. The WT sequence is shown above. Deletions (denoted with “Δ” and the number of base pairs) are identified

**Fig. 4:**
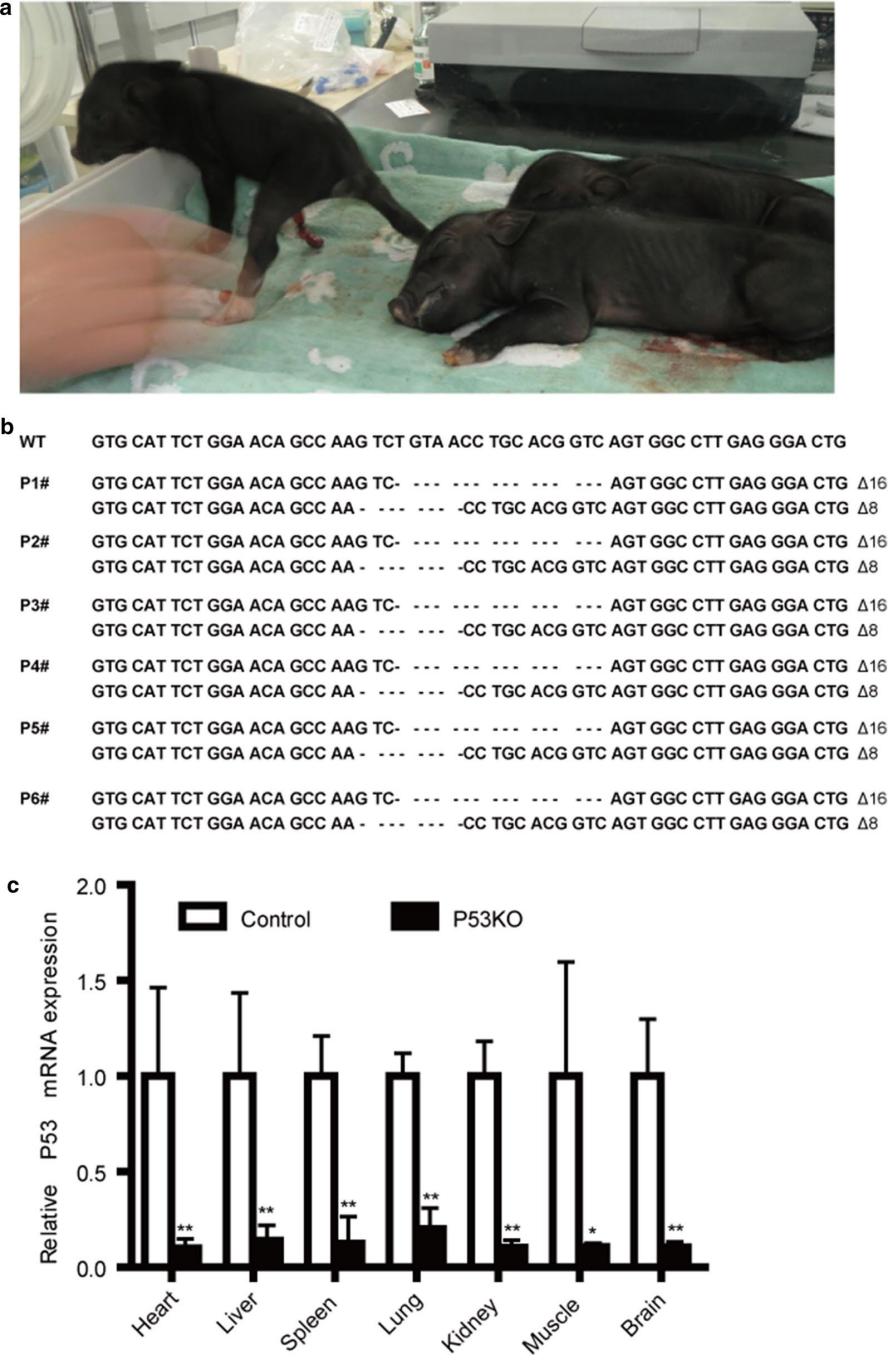
Identification of transgenic piglets. **a** Obtained partly P53 KO piglets. **b** The sequences of the P53 mutation in cloned piglets. The WT sequence is shown above. Deletions (denoted with “Δ” and the number of base pairs) are identified. **c** The relative expression levels of *P53* mRNA in the different tissues from P53 KO and WT piglets. The relative expression levels of *P53* mRNA in brain, muscle, kidney, heart, liver and kidney tissues of P53 KO and WT piglets were measured using q-PCR. Expression of the *GAPDH* gene was used to normalize the values of *P53*. * *p* < 0.05 and ** *p* < 0.01 denote significant differences in P53 KO piglets compared to WT piglets

One homozygous mutant cell colony (C12#) was used as donor cells for SCNT. We produced 2068 reconstructed embryos by SCNT. The cleavage and blastocyst formation rates of the embryos were 76.3% and 26.7%, respectively (Table 1). A total of 1705 cloned embryos were transferred into five surrogates (Table 2), and three of them became pregnant. Five morphologically normal fetuses (Fig. 3a and Table 2) were obtained from one recipient after 38 days, and their primary fibroblasts were isolated and cultured. Sequencing analysis confirmed five fetuses as P53 knockout fetuses in accordance with the C12# colony (Fig. 3b). Six live piglets and one dead piglet (Fig. 4a and Table 2) were obtained from two recipients after 111 or 112 days. The DNA sequencing showed that all six live piglets carried biallelic mutations in the P53 and the genotype was consistent with the donor cells derived from C12# colony (Fig. 4b). Four piglets died immediately after birth and the remaining two died at approximately 2 and 5 months (Table 3). The mRNA expression level in the various tissues of the P53 biallelic knockout and WT piglets was detected, and the results showed that P53 mRNA expression was significantly reduced compared to the WT, which confirmed the disruption of P53 expression (Fig. 4c).

**Table 1:** Developmental competence of reconstructed embryos after fusion and electrical activation

No. of embryos (repeat)	Cleavage (%)	Blastocyst (%)	No. of cells in blastocysts
1356 (6)	846 (76.3 ± 4.0)	367 (26.7 ± 3.9)	54.6 ± 13.6

The percentages are expressed as the Mean ± SD

**Table 2:** Nuclear transfer efficiencies of SCNT

Donor cells	Recipients	Transferred embryos	Days of pregnancy (d)	The pregnancy rate	Offspring (stillborn/aborted)	Mutant piglets or fetuses
	1	449	–	60%	–	–
	2	277	–		–	–
C12#	3	312	38 (cesarean)		5 (fetuses)	5
	4	348	112 (cesarean)		2	2
	5	319	111 (cesarean)		5 (1 dead)	5

**Table 3:** The survival time of the P53 KO piglets

No. of the P53 KO piglets	Survival time of the P53 KO piglets
P1#	154 days
P2#	89 min
P3#	71 days
P4#	85 min
P5#	144 min
P6#	202 min

### Functional inactivation of the porcine P53 protein knockout

**Fig. 5:**
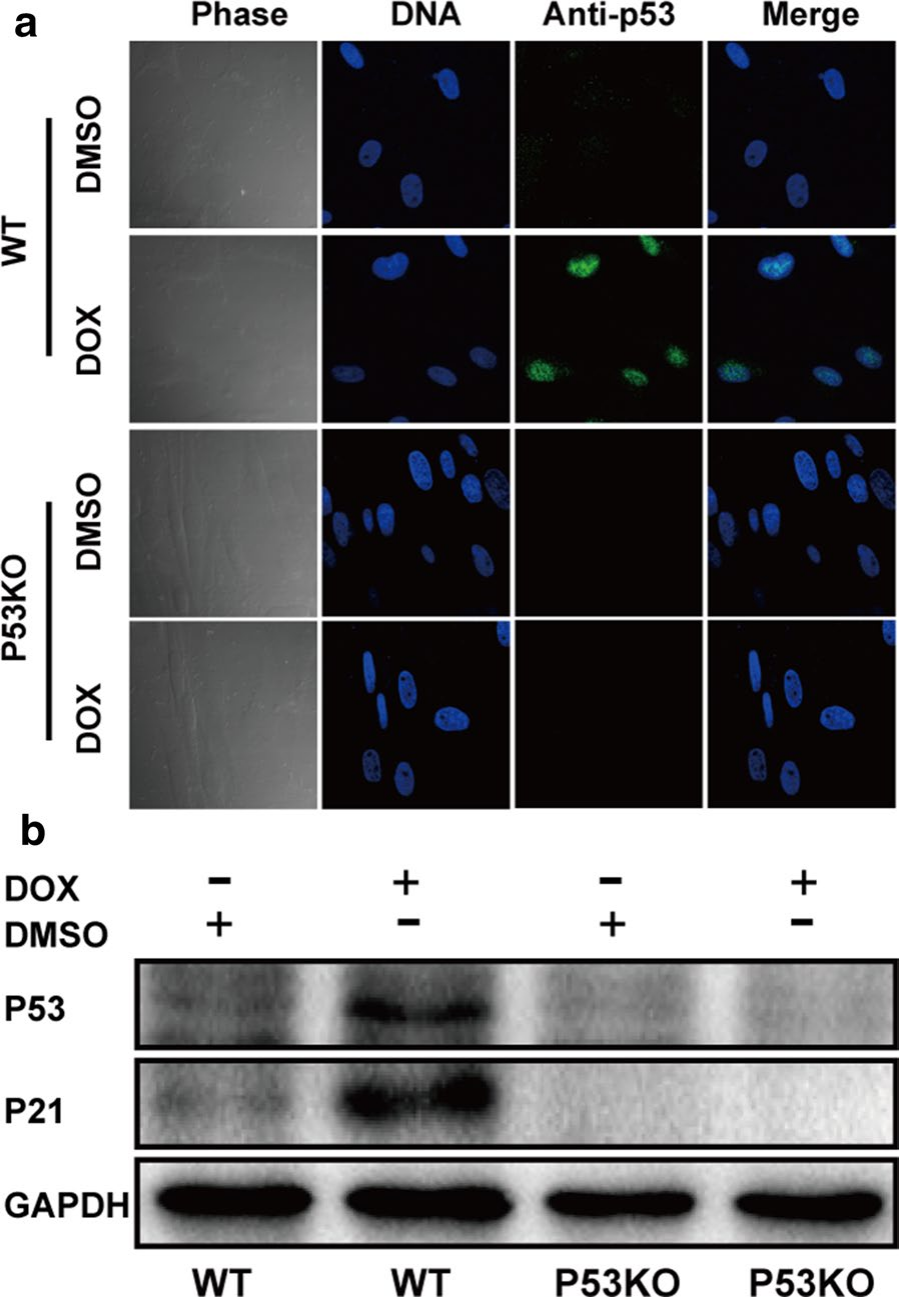
Phenotype detections. **a** The intracellular localization of P53 was analyzed using confocal fluorescence microscopy. The fibroblast cells from P53 KO piglets and WT piglets were treated with DOX at 100 µM for 24 h and stained with Hoechst 33342 (blue) and an anti-P53 antibody (green). **b** The fibroblast cells were treated as described above and protein expression levels were examined by Western blotting. P53 and P21 protein expression in the treated fibroblasts are shown in cropped blots using an anti-P53 and anti-P21 monoclonal antibody. Anti-GAPDH served as a loading control

It was anticipated that no P53 protein would be produced in P53 knockout pigs. Thus, we tested whether the porcine P53 knockout behaves like its human and mouse orthologs by analyzing its expression and activity in primary pig fetal fibroblasts in the absence or presence of DOX (doxorubicin)-induced DNA damage. As shown in Fig. 5a, comparable levels of P53 protein were induced by doxorubicin in WT fibroblasts, but not in P53 KO fibroblasts. Consistently, western blotting analysis indicated that DNA damage caused by doxorubicin treatment induced the robust protein expression of P53 and P21 in WT fibroblasts, but not in P53 KO fibroblasts (Fig. 5b). Together, these findings confirmed that the porcine P53 protein was functionally defective in P53 KO *Diannan* miniature pigs, similar to its human and mouse counterparts.

## Discussion

Mice or pigs with P53 mutations are usually generated by the Cre/loxP system and recombinant adeno-associated virus [18, 19, 30]. However, these methods have an extremely low efficiency because of their dependence on homologous recombination [20, 31]. Recently, it has been reported that the TALEN system exhibits high targeting efficiency and specificity [32–34]. To date, TALENs have been successfully applied for efficiently targeting genes as well as generating several animal models [35–38]. Furthermore, our previous study showed the TALEN plasmid DNA editing in sheep did not observe detectable off-target effects using whole-genome sequencing [28]. This result suggests that TALEN plasmid DNA editing in *Diannan* miniature pigs will also have no off-target effects. Therefore, in this study, we used TALEN technology to target genes in porcine somatic cells followed by SCNT to produce P53 KO pigs. As expected, the TALEN targeting efficiency in PFFs was up to 42%. Five were biallelic knockouts, which indicated that TALENs was a more efficient method for disrupting the P53 allele in porcine fibroblast cells. We performed a total of five embryo transfers, resulting in seven P53 biallelic KO piglets from two full-term pregnancies (Table 2). In addition, we collected five P53 biallelic KO fetuses from one pregnant sow to establish primary cell lines for future use. The pregnancy rate was 60%, similar with the results of Sieren et al. [19]. Furthermore, the porcine fibroblasts of the P53 biallelic knockout did not respond to Dox, which demonstrated that the P53 gene was dysfunctional in these pigs. Recently, an improved CRISPR/Cas9 system enabled more efficient and more precise gene editing, including a point mutation [39–42], which is promising for mimicking various human P53 disruptions. Whether it can produce the models simulating various human P53 disruptions with the CRISPR/Cas9 system need to be further investigated.

In previous studies, a mouse model with P53 mutation or deficiency was developed for the study of human tumors [12, 43, 44]. P53-mutant mice are usually used to investigate Li-Fraumeni syndrome, which may cause a variety of tumors, including sarcomas, breast cancers, brain tumors and adrenocortical carcinomas [45]. However, various types of P53 mutation could have different effects on animals, including tumorigenesis and anti-tumorigenesis [44]. In P53-deficient homozygote mice, the tumors most frequently observed are malignant lymphomas [30, 43, 46, 47]. While mouse tumor models are widely used, their small size and short lifespan preclude some applications in preclinical studies. Pigs are increasingly important in biomedicine and offer valuable complementary resources for cancer research [48–51]. Recently, P53-mutant pigs also have been generated, which mostly mimic the mutation of the R175H locus in the human P53 gene, and lymphomas and osteosarcoma have been observed in these pig models [18, 19]. However, the tumorigenesis types have not been reported in P53-deficient pigs to date.

In this study, of the six live P53 biallelic knockout *Diannan* miniature pigs, four died immediately after birth, and the remaining two died at approximately 2 and 5 months. These P53 KO pigs were examined by necropsy and hematoxylin–eosin (HE) staining and had no evidence of tumors (data not shown). A recent study reported that although P53 inactivation in pigs was sufficient for spontaneous tumorigenesis, there was also no evidence of tumors or other abnormalities in the animals younger than 16 months [18]. Sieren et al. [19] also reported that necropsy did not reveal any discreet tumors in newborn P53-mutated piglets, although those pigs that reached sexual maturity developed lymphomas and osteogenic and renal tumors. Thus, we infer that our pigs might have not reached the age of tumorigenesis before their death. A lack of samples also might have led to the failure to find tumors in P53 biallelic KO *Diannan* miniature pigs. More P53 biallelic KO pigs must be generated, and the tumorigenesis types of P53 KO pigs require further investigation.

## Conclusions

The combination of TALEN gene editing technology and SCNT is effectively used for generating P53 biallelic KO *Diannan* miniature pigs. Although these genetically engineered *Diannan* miniature pigs had no tumorigenic signs, the P53 gene was dysfunctional in these pigs. We believe that these pigs will provide powerful new resources for preclinical oncology and basic cancer research.

## Additional file

**Additional file 1: Table S1.** Primer sequences for the genes.

## Abbreviations

rAAV: recombinant adeno-associated virus
TALENs: transcription activator-like effector nucleases
DSB: double-strand break
SCNT: somatic cell nuclear transfer
SSA: single strand annealing
PFFs: pig fetal fibroblasts
T7EI: T7 endonuclease I
q-PCR: quantitative-polymerase chain reaction
PVDF: polyvinylidene difluoride
ECL: chemiluminescence
DOX: doxorubicin
