# Supplementary material for: Efficient generation of P53 biallelic knockout Diannan miniature pigs via TALENs and somatic cell nuclear transfer
Source: J Transl Med. 2017 Nov 3;15:224. doi: 10.1186/s12967-017-1327-0 (PMC5670695; doi:10.1186/s12967-017-1327-0)
Supplement: Supplementary file 1 — Additional file 1: Table S1. Primer sequences for the genes. [file 12967_2017_1327_MOESM1_ESM.docx]

**Table S1. Primer sequence for the genes listed below.**

| **Gene** | **Primer sequence (5’ to 3’)** |
| --- | --- |
| GAPDH | F ATCAAGAAGGTGGTGAAGCAC |
|  | R CAGCATCAAAAGTGGAAGAGTG |
| P53 | F CACTGGATGGCGAGTATTTCAC |
|  | R CGCAGTCTGGGCATCCTTC |
